# Supplementary material for: Into the Dark: Exploring the Deep Ocean with Single-Virus Genomics
Source: Viruses. 2022 Jul 21;14(7):1589. doi: 10.3390/v14071589 (PMC9322844; doi:10.3390/v14071589)
Supplement: Supplementary file 1 [file viruses-14-01589-s001.zip › viruses-1760592-supplementary figures.pdf]

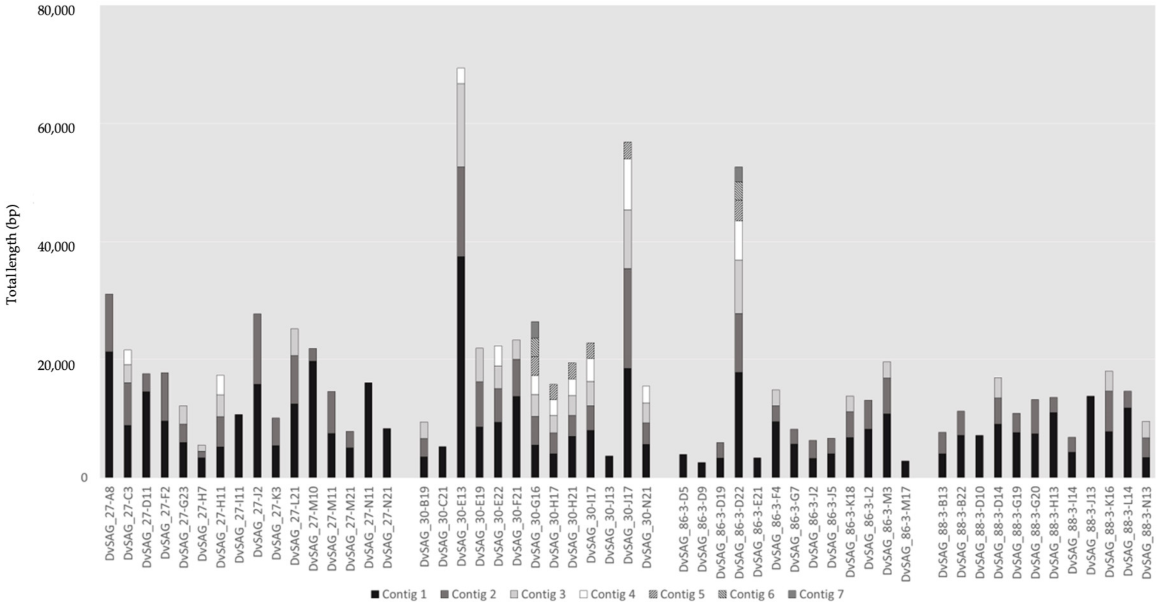

Supplementary Figure S1. vSAG contigs information Each bar represent one of the sequenced vSAGs with contigs longer than 2500 bp. Every color within each bar corresponds with different contigs from the same vSAG.

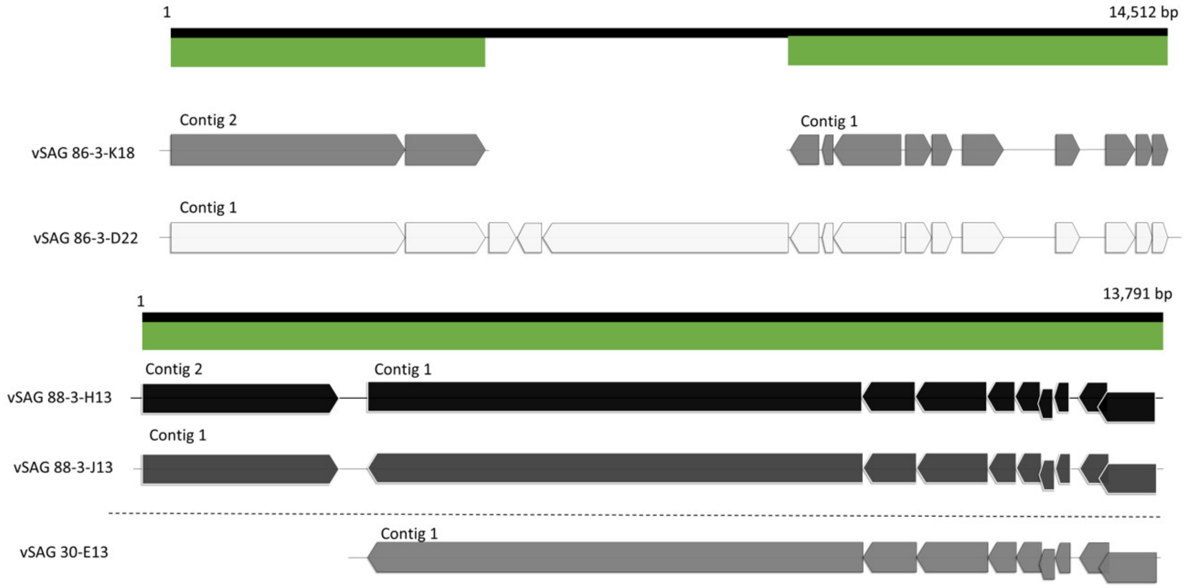

Supplementary Figure S2. Genome alignment between related vSAGs. Green color indicates identical nucleotide positions along the alignment.
